# Biased under-reporting of research reflects biased under-submission more than biased editorial rejection

**DOI:** 10.12688/f1000research.2-1.v1

**Published:** 2013-01-02

**Authors:** Iain Chalmers, Kay Dickersin

**Affiliations:** 1 James Lind Initiative, Summertown Pavilion, Middle Way, Oxford, OX2 7LG, UK; 2 Center for Clinical Trials, Johns Hopkins Bloomberg School of Public Health, 615 N. Wolfe St. Rm E6152, Baltimore, Maryland, 21205, USA

## Abstract

Stephen Senn challenges Ben Goldacre’s assertion in ‘Bad Pharma’ that biased editorial acceptance of reports with ‘positive’ findings is not a cause of biased under-reporting of research. We agree with Senn that biased editorial decisions may contribute to reporting bias, but Senn ignores the evidence that biased decisions by researchers to submit reports for possible publication are the main causes of the problem.

Stephen Senn challenges Ben Goldacre’s assertion in ‘ *Bad Pharma*’ ^1^ that biased editorial acceptance of reports with ‘positive’ findings is not a cause of biased under-reporting of research, and concludes that "the prospects for disentangling cause and effect when it comes to publication bias are not great" ^2^. Senn apparently overlooks the studies – including controlled experiments - which have investigated reporting biases. These are summarised in an article ^3^ from which the following is an excerpt:

“ ***Who is responsible for biased reporting of clinical research?***

*Reporting bias can be due to researchers and sponsors failing to submit study findings for publication, or due to journal editors and others rejecting reports for publication. Numerous surveys of investigators have left little doubt that almost all failure to publish is due to the failure of investigators to submit reports for publication* ^4, 5^, *with only a small proportion of studies remaining unpublished because of rejection by journals* ^6^, *although positive-outcome bias has been demonstrated among peer reviewers* ^7^. *Qualitative studies of editorial discussion indicate that a study’s scientific rigour is the area of greatest concern* ^8^. *Researchers report that the reason they do not write up and submit reports of their research for publication is usually because they are "not interested" in the results ("editorial rejection by journals" is only rarely given as a cause of failure to publish). Even those investigators who have initially published their results as (conference) abstracts are less likely to submit their findings for full publication unless the results are ‘significant’* ^9^.

*Investigations of biased reporting of research began with surveys of journal articles, which revealed improbably high proportions of published studies showing statistically significant differences* ^10– 14^. *Subsequent surveys of authors and peer reviewers showed that research that had yielded ‘negative’ results was less likely than other research to be submitted or recommended for publication* ^15– 18^. *These findings have been reinforced by the results of experimental studies, which showed that studies with no reported statistically significant differences were less likely to be accepted for publication* ^7, 19– 21^".

Senn’s use of the term ‘publication bias’ in his commentary suggests that he is restricting it to editorial bias whereas, as indicated above, the origins of reporting bias are largely due to researchers’ decisions not to submit, not editorial decisions not to accept. The analyses of observational data cited by Ben Goldacre in his book ‘ *Bad Pharma*’ ^1^ do not detect editorial bias, but neither do they support a confident conclusion that no editorial bias exists. However, we believe Goldacre is correct to castigate researchers and research sponsors as being more culpable than editors in betraying their responsibility to the patients who have participated in trials.

The controlled experiments suggest that it is the results of studies, not their quality, that predisposes them to editorial bias. Senn believes that any editorial bias that exists can be ‘very plausibly explained’ by preferential publication of ‘positive’ studies, and that it "seems plausible that higher quality studies are more likely to lead to a positive result". Unless he is using the word ‘positive’ to mean something other than ‘a beneficial effect’, however, Senn appears to be overlooking substantial evidence challenging the plausibility of his belief (see, for example, reference ^22^). Given the estimated likelihood of new treatments proving superior to standard treatments ^23^ it surprises us that, "as a statistician" Senn would find this evidence "unpalatable".
