# Supplementary material for: Biased under-reporting of research reflects biased under-submission more than biased editorial rejection
Source: F1000Res. 2013 Jan 2;2:1. [Version 1] doi: 10.12688/f1000research.2-1.v1 (PMC3782352; doi:10.12688/f1000research.2-1.v1)
Supplement: Peer review history

# Peer review: Referee response for version 1

**Published:** 2013-01-09

**Authors:** Steven A Julious

**Affiliations:** 1 Medical Statistics Group, School of Health and Related Research, University of Sheffield, Sheffield, UK

I would just put one anecdotal observation and that is of second studies that replicate the findings of a study published in a journal. An editor may turn down the second study as 'nothing new' is being said although most would argue replication to be important.

I have read this submission. I believe that I have an appropriate level of expertise to confirm that it is of an acceptable scientific standard.

# Peer review: Referee response for version 1

**Published:** 2013-01-08

**Authors:** Luigi Naldi

**Affiliations:** 1 Department of Dermatology, Centro Studi GISED, Bergamo, Italy

I have read this submission. I believe that I have an appropriate level of expertise to confirm that it is of an acceptable scientific standard.

# Peer review: Referee response for version 1

**Published:** 2013-01-08

**Authors:** Riekie de Vet

**Affiliations:** 1 EMGO Institute for Health and Care Research, VU University Medical Center, Amsterdam, Netherlands

The authors comment on a article by Stephen Senn who questions Ben Goldacre’s assertion in the book “Bad Pharma” that editorial process is not the main cause of publication bias. They present a large amount of evidence from the literature that researchers are the main cause of publication bias by selectively submitting paper for publication. 

They provide a lot of convincing information in this short reaction. However, some sentences are very difficult to read. Especially for readers who haven’t read the book by Goldacre, the comment by Senn, and some of the other references. I had to reread the first sentence about five times before I understood. The sentence is especially difficult to read because there is a double negation. Splitting the sentence in the statement of Ben Goldacre and the comment of Stephen Senn may help.  Also the last sentence of the comment is difficult to understand, especially when the reader is unaware of the conclusion of reference 23. 

The second part of the citation of Goldacre “the prospects for disentangling cause and effect when it comes to publication bias are not great” is difficult to understand and, as far as I can see, does not come back in the comment. Consider whether that part can be omitted, or refer to it again at the end of the comment. 

The last section starts with ‘The controlled experiments’. It is not clear to which experiments this refers. To ‘studies – including controlled experiments ‘mentioned in the first section?  

In conclusion, this is a very important and informative comment. However, the readability should be improved in order to make it better understandable for readers who have not read all previous papers.

I have read this submission. I believe that I have an appropriate level of expertise to confirm that it is of an acceptable scientific standard.
